# Simplified Procedure to Determine the Cohesive Material Law of Fiber-Reinforced Cementitious Matrix (FRCM)–Substrate Joints

**DOI:** 10.3390/ma17071627

**Published:** 2024-04-02

**Authors:** Francesco Focacci, Tommaso D’Antino, Christian Carloni

**Affiliations:** 1 eCampus University, 22060 Novedrate, Italy; francesco.focacci@uniecampus.it; 2 Department of Architecture, Built Environment and Construction Engineering, Politecnico di Milano, 20133 Milan, Italy; 3 Department of Civil and Environmental Engineering, Case Western Reserve University, Cleveland, OH 44106, USA; christian.carloni@case.edu

**Keywords:** cohesive material law (CML), FRCM, TRM, calibration, direct shear test

## Abstract

Fiber-reinforced cementitious matrix (FRCM) composites have been largely used to strengthen existing concrete and masonry structures in the last decade. To design FRCM-strengthened members, the provisions of the Italian CNR-DT 215 (2018) or the American ACI 549.4R and 6R (2020) guidelines can be adopted. According to the former, the FRCM effective strain, i.e., the composite strain associated with the loss of composite action, can be obtained by combining the results of direct shear tests on FRCM–substrate joints and of tensile tests on the bare reinforcing textile. According to the latter, the effective strain can be obtained by testing FRCM coupons in tension, using the so-called clevis-grip test set-up. However, the complex bond behavior of the FRCM cannot be fully captured by considering only the effective strain. Thus, a cohesive approach has been used to describe the stress transfer between the composite and the substrate and cohesive material laws (CMLs) with different shapes have been proposed. The determination of the CML associated with a specific FRCM–substrate joint is fundamental to capture the behavior of the FRCM-strengthened member and should be determined based on the results of experimental bond tests. In this paper, a procedure previously proposed by the authors to calibrate the CML from the load response obtained by direct shear tests of FRCM–substrate joints is applied to different FRCM composites. Namely, carbon, AR glass, and PBO FRCMs are considered. The results obtained prove that the procedure allows to estimate the CML and to associate the idealized load response of a specific type of FRCM to the corresponding CML. The estimated CML can be used to determine the onset of debonding in FRCM–substrate joints, the crack number and spacing in FRCM coupons, and the locations where debonding occurs in FRCM-strengthened members.

## 1. Introduction

**Figure 1:**
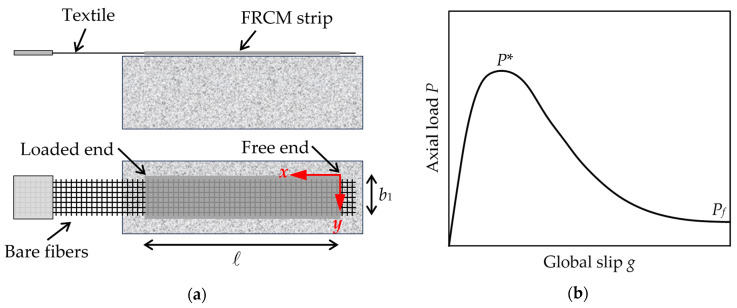
(**a**) Sketch of a specimen used in single-lap direct shear tests; (**b**) Idealized load response obtained by a direct shear test of a FRCM–substrate joint that failed due to debonding at the matrix–fiber interface.

Fiber-reinforced cementitious matrix (FRCM) composites have attracted the interest of the civil engineering industry as an alternative to fiber-reinforced polymer (FRP) composites for strengthening/retrofitting existing concrete and masonry members. FRCMs comprise open mesh textiles embedded within an inorganic matrix. The textile can be made of various types of fiber, e.g., carbon, basalt, glass, and polyparaphenylene benzobisoxazole (PBO), whereas the matrix is usually a cement- or a lime-based mortar. FRCMs are externally bonded (EB) to existing concrete and masonry members and can be used to improve bending [1,2,3,4] and shear strengths [5,6,7,8], as well as the axial compressive capacity of predominantly axially-loaded members [9,10,11,12]. EB FRCM reinforcement generally fails due to debonding of the composite at the FRCM–substrate interface, with or without damage of the substrate, or at the matrix–fiber interface [13]. Understanding the FRCM bond behavior is thus fundamental to properly assess the reinforcement effectiveness. The bond between FRCM and different substrates was studied using direct shear tests and small-scale beam tests [14,15,16,17,18]. In the single-lap direct shear test set-up recommended by the Italian [19] and European [20] acceptance criteria for FRCM composites, the FRCM strip is applied to one face of the substrate block and a portion of textile is left bare at the loaded end (beyond the bonded area) to be gripped and pulled by the testing machine, while the substrate is constrained (Figure 1a). During this test, the load *P* applied to the FRCM textile and the relative displacement between the textile and the substrate at the composite loaded end, named global slip *g*, are measured. An idealized load response obtained by the direct shear test of an FRCM–substrate joint that failed due to debonding at the matrix–fiber interface is shown in Figure 1b. This load response comprises an initial ascending branch and a subsequent descending branch that ends with a constant applied load *P_f_*. *P_f_* is provided by friction at the matrix–fiber interface after debonding has occurred along the entire bonded length and was observed for different FRCM composites [21,22]. The presence of friction is responsible for a peak load P* higher than that associated with the onset of debonding, provided that the bonded length is greater than the minimum length needed to fully develop the bond stress transfer mechanism, i.e., the effective bond length [23].

The bond behavior of an FRCM–substrate joint can be described using the differential equation [23]:

(1) $$\frac{d^{2}s(x)}{dx^{2}}-\frac{p_{f}}{E_{f}A_{f}}\tau_{zx}(x)=0$$

where *s*(*x*) is the matrix–fiber slip (the reference system is shown in Figure 1a), τ*_zx_*(*x*) is the matrix–fiber shear stress, *p_f_* is the matrix–fiber contact perimeter, *E_f_* is the textile elastic modulus, and *A_f_* is the textile cross-sectional area. In the remainder of the paper, only shear stresses in the direction of the load will be considered and the subscript *zx* will be omitted for the sake of brevity. Equation (1) is based on the assumption of a pure Mode-II loading condition at the interface where debonding occurs. This assumption, which is often adopted to describe the results of single-lap direct shear tests, is supported by the presence of the matrix layer that covers the textile in FRCM composites, which contrasts the effect of a possible Mode-I loading component.

**Figure 2:**
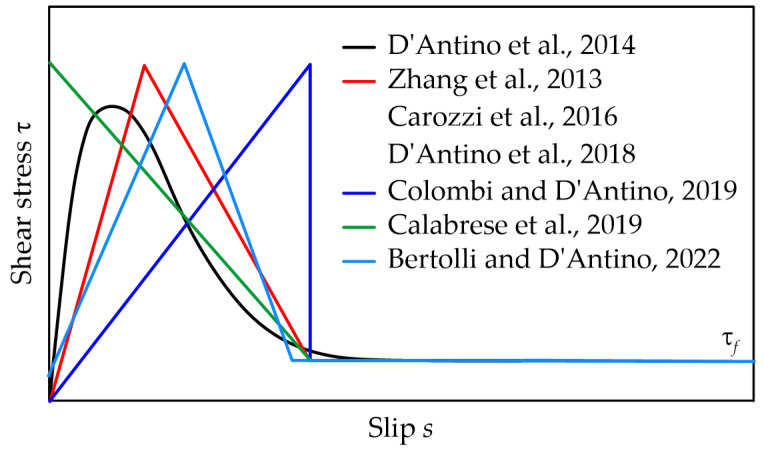
Shear stress–slip curves proposed in the literature [23,25,26,27,28,29,30].

Once the relationship between the matrix–fiber shear stress and slip, i.e., the interfacial cohesive material law (CML), is known, Equation (1) can be used to describe the stress transfer mechanism along the joint bonded length and study the contribution of EB FRCM strips to the capacity of strengthened members [24]. Various shapes of the shear stress–slip relationship were proposed in the literature (Figure 2). Among them, an exponential CML was proposed in [25] to describe the matrix–fiber bond behavior of PBO FRCM-concrete joints. Different piece-wise functions were also proposed. A trilinear CML was used in [26,27,28], while an elasto–brittle relationship and a rigid–cohesive CML were used in [29] and [30], respectively. Finally, a rigid–trilinear CML was proposed to obtain finite values of the effective bond length in PBO FRCM–substrate joints [23]. These shapes can be adopted to describe the CML associated with interfaces with different mechanical properties.

A procedure to calibrate a trilinear CML (see Figure 2) using the load response obtained with direct shear tests of FRCM–substrate joints was proposed by the authors in [31]. Since FRCM composites can be manufactured using textiles with different fibers, layouts, and types of matrixes and can be applied to various substrates, FRCM–substrate joints often have a peculiar behavior. To verify the capability of the procedure proposed in capturing the complex behavior shown by various FRCM–substrate joints, in this paper it was applied to carbon, AR glass, and PBO FRCM composites applied to concrete and masonry substrates.

## 2. Calibration of the Proposed Trilinear CML

The proposed trilinear CML consists of a linear ascending branch up to the slip *s*_0_ and maximum shear stress τ*_m_*, followed by a linear descending branch up to the slip *s_f_* and a constant branch, corresponding to the friction shear stress τ*_f_*. τ*_f_* could also be equal to zero. The trilinear CML proposed can be calibrated starting from the applied load *P*–global slip *g* response obtained with a direct shear test (experimental *P*–*g* response) following 6 steps. These steps were previously described by the authors in [31] and are recalled here for the sake of clarity. The experimental *P*–*g* response consists of a set of applied forces $P_{j}$ (j=1,2,…,N) and corresponding measured global slips $g_{j}$ (j=1,2,…,N).

**Step 1**. In general, experimental data present small oscillations that can affect the calibration procedure proposed. In the first step, Equations (2)–(4) were employed to reduce these oscillations and obtain a set of global slips ${\bar{g}}_{k}$ and a set of applied load ${\bar{P}}_{k}$ ($k=1,2,\ldots ,\bar{N}$) of $g_{j}$ and $P_{j}$ (j=1,2,…,N), respectively:

(2) $$J_{1}=1\;J_{i}=\mathrm{int}\left[\frac{N}{\bar{N}}\left(i-1\right)\right]\;\;\;\;\;\;i=2,3,\ldots ,\bar{N}+1$$

(3) $${\bar{g}}_{k}=\frac{1}{J_{k+1}-J_{k}}\sum_{i=J_{k}}^{J_{k+1}-1}g_{i}\;\;\;\;\;k=1,2,\ldots ,\bar{N}$$

(4) $${\bar{P}}_{k}=\frac{1}{J_{k+1}-J_{k}}\sum_{i=J_{k}}^{J_{k+1}-1}P_{i}\;\;\;\;\;k=1,2,\ldots ,\bar{N}$$

where $\bar{N}$ is the number of elements in ${\bar{g}}_{k}$ and ${\bar{P}}_{k}$, which can be determined using a trial and error procedure until a satisfactory solution is obtained, and *int*(*Z*) denotes the positive integer number nearest to the rational number *Z*.

**Step 2**. The friction shear stress τ*_f_* (see Figure 2), if any, can be determined from the approximately constant applied load *P_f_* at the end of the *P*–*g* response of specimens that showed matrix–fiber debonding. According to the procedure proposed, *P_f_* is the average applied load for global slips higher than *g_f_*, which is the global slip associated with a slope of the *P*–*g* response lower than a certain $P_{f}^{\prime }$ that needs to be defined by the user:

(5) $$P_{f}=\frac{1}{\bar{N}-k_{f}+1}\sum_{k=k_{f}}^{\bar{N}}{\bar{P}}_{k}$$

with

(6) $$k\geq k_{f}\Rightarrow \left|P_{k}^{\prime }\right|\leq P_{f}^{\prime }$$

(7) $$P_{k}^{\prime }=\frac{{\bar{P}}_{k+1}-{\bar{P}}_{k}}{{\bar{g}}_{k+1}-{\bar{g}}_{k}}\;k=1,2,\ldots ,\bar{N}-1$$

Once *P_f_* is known, τ*_f_* can be obtained as the constant shear stress acting at the matrix–fiber interface along the bonded length ℓ (Figure 1):

(8) $$\tau_{f}=\frac{P_{f}}{\ell p_{f}}$$

**Figure 3:**
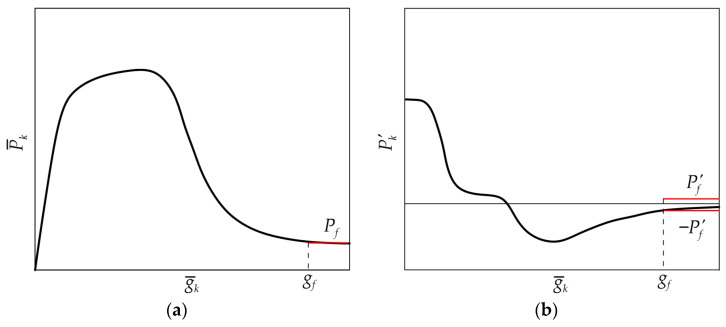
Idealized (**a**) ${\bar{P}}_{k}-{\bar{g}}_{k}$ and (**b**) corresponding $P_{k}^{\prime }-{\bar{g}}_{k}$ responses.

Figure 3a shows an idealized ${\bar{P}}_{k}-{\bar{g}}_{k}$ curve with the indication of *g_f_* and of the average applied load for ${\bar{g}}_{k}\geq g_{f}$, whereas Figure 3b shows the corresponding $P_{k}^{\prime }-{\bar{g}}_{k}$ curve with the indication of $g_{f}={\bar{g}}_{k_{f}}$.

**Step 3**. The slope *h* of the ascending branch of the CML can be computed from the slope *p*_0_ of the ascending branch of the *P*–*g* curve. The slope of the ascending branch of the CML can be computed as

(9) $$p_{0}=\frac{P_{2}-P_{1}}{g_{2}-g_{1}}$$

where *P*_1_ and *P*_2_ are the applied loads associated with 0.1P* and 0.5P* and *g*_1_ and *g*_2_ the corresponding global slips extracted from the $P_{j}$−$g_{j}$ response. It should be noted that this method works provided that *g*_1_ and *g*_2_ are smaller than *s*_0_, which should be verified at the end of the procedure. If the slip *s*_0_ resulting from the procedure is smaller than *g*_2_, the procedure can be repeated using a *P*_2_ smaller than 0.5P*. The slope *h* of the ascending branch of the trilinear CML can be obtained as

(10) $$h=\frac{p_{0}^{2}}{A_{f}E_{f}p_{f}}$$

**Step 4**. In this step, the oscillation of the ascending and part of the descending branches of the *P*–*g* response (note that the descending branch is considered only to ensure that the stress transfer mechanism is fully established) are reduced via Equations (11)–(13). In particular, global slips ${\tilde{g}}_{k}$ and corresponding applied loads ${\tilde{P}}_{k}$ ($k=1,,\ldots ,\tilde{N}+1$) were obtained:

(11) $$n_{g}=\mathrm{int}\left(\frac{j_{\max}+\tilde{N}}{\tilde{N}}\right)$$

(12) $${\tilde{g}}_{k}=\frac{1}{n_{g}}\sum_{i=\left(k-1\right)n_{g}+1}^{n_{g}k}g_{i}\;\;\;\;\;k=1,2,\ldots ,\tilde{N}+1$$

(13) $${\tilde{P}}_{k}=\frac{1}{n_{g}}\sum_{i=\left(k-1\right)n_{g}+1}^{n_{g}k}P_{i}\;\;\;\;\;k=1,2,\ldots ,\tilde{N}+1$$

where $\tilde{N}+1$ is the number of ${\tilde{P}}_{k}$ and ${\tilde{g}}_{k}$ points obtained and *j*_max_ is the index of the maximum load in the set of *P_j_*.

**Step 5**. This step allows for identifying the slip *s_f_* at the onset of debonding. Equation (14) is employed to compute the shear stress ${\tilde{\tau }}_{k}$ associated with each ${\tilde{g}}_{k}$ ($\;k=1,2,\ldots ,\tilde{N}$):

(14) $${\tilde{\tau }}_{k}=\frac{1}{2p_{f}E_{f}A_{f}}\frac{{\tilde{P}}_{k+1}^{2}-{\tilde{P}}_{k}^{2}}{{\tilde{g}}_{k+1}-{\tilde{g}}_{k}}\;\;\;\;\;k=1,2,\ldots ,\tilde{N}$$

**Figure 4:**
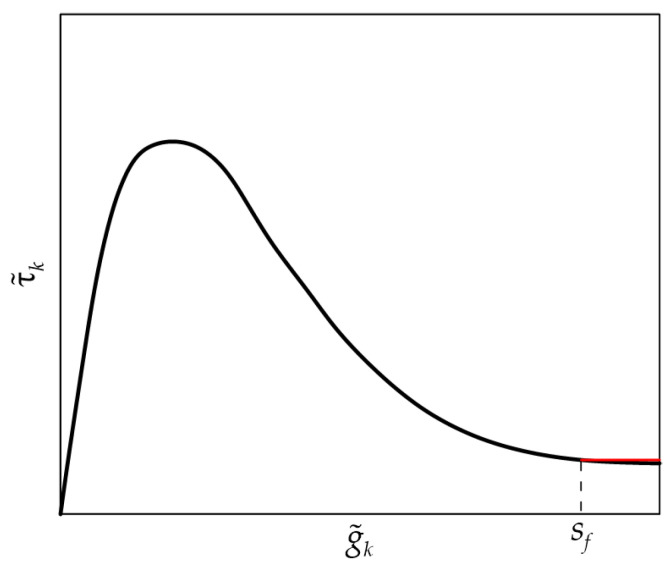
Idealized ${\tilde{\tau }}_{k}$−${\tilde{g}}_{k}$ curve and slip *s_f_* at the beginning of the friction branch (the constant friction branch is indicated with a red line).

The ${\tilde{\tau }}_{k}$−${\tilde{g}}_{k}$ response represents the experimental CML obtained from the ${\tilde{P}}_{k}$−${\tilde{g}}_{k}$ response. *s_f_* is the slip corresponding to τ*_f_* in the ${\tilde{\tau }}_{k}$−${\tilde{g}}_{k}$ response and can be computed as

(15) $$s_{f}=\frac{{\tilde{g}}_{k_{fr}-1}+{\tilde{g}}_{k_{fr}}}{2}$$

where *k_fr_* is the minimum index *k* such that ${\tilde{\tau }}_{k_{fr}-1}>\tau_{f}$ and ${\tilde{\tau }}_{k_{fr}}<\tau_{f}$. Figure 4 shows the ${\tilde{\tau }}_{k}$−${\tilde{g}}_{k}$ relationship provided by Equation (14) considering the ${\tilde{P}}_{k}$−${\tilde{g}}_{k}$ response obtained with the idealized response of Figure 3a, where the horizontal constant branch starting at *s_f_*, computed by Equation (15), is indicated with a red line.

It should be noted that Equation (14) was obtained from the well-known fracture mechanics relationship in Equation (16) [32], which is valid only if the free end slip is null.

(16) $$P\left(g\right)=\sqrt{2p_{f}E_{f}A_{f}\int_{0}^{g}\tau \left(s\right)\;ds}$$

**Step 6**. The fracture energy *G_F_*, which is the area below the CML from *s* = 0 to *s* = *s_f_*, can be obtained by applying the trapezoidal rule to the ${\tilde{\tau }}_{k}$−${\tilde{g}}_{k}$ relationship:

(17) $$G_{F}=\frac{s_{f}}{k_{fr}}\left(\frac{{\tilde{\tau }}_{1}+\tau_{f}}{2}+\sum_{k=1}^{k_{fr}-1}{\tilde{\tau }}_{k}\right)$$

**Step 6a**. Since the applied load is assumed to be evenly distributed across the composite width, i.e., there is no width effect, the fracture energy *G_F_* can be obtained, as an alternative to the procedure in Step 6, by rearranging Equation (16) and considering the debonding load *P_deb_*, i.e., the applied load associated with the onset of debonding (*g* = *s_f_*):

(18) $$P_{deb}=\frac{{\tilde{P}}_{k_{fr}-1}+{\tilde{P}}_{k_{fr}}}{2}$$

(19) $$G_{F}=\frac{P_{deb}^{2}}{2p_{f}E_{f}A_{f}}$$

**Figure 5:**
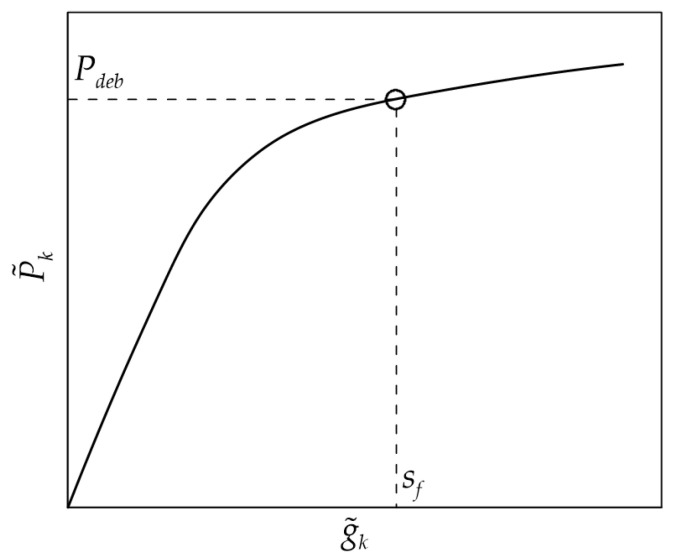
Debonding load *P_deb_* on the *P*–*g* response obtained from Figure 3a.

Figure 5 shows the identification of *P_deb_* on the idealized load response of Figure 3a.

**Step 7**. The trilinear CML peak shear stress τ*_m_* and corresponding slip *s*_0_ can be obtained from the fracture energy *G_F_*, slope of the ascending branch *h*, shear stress at the onset of debonding τ*_f_*, and corresponding slip *s_f_*:

(20) $$s_{0}=\frac{2G_{F}-s_{f}\tau_{f}}{hs_{f}-\tau_{f}}$$

(21) $$\tau_{m}=hs_{0}$$

**Figure 6:**
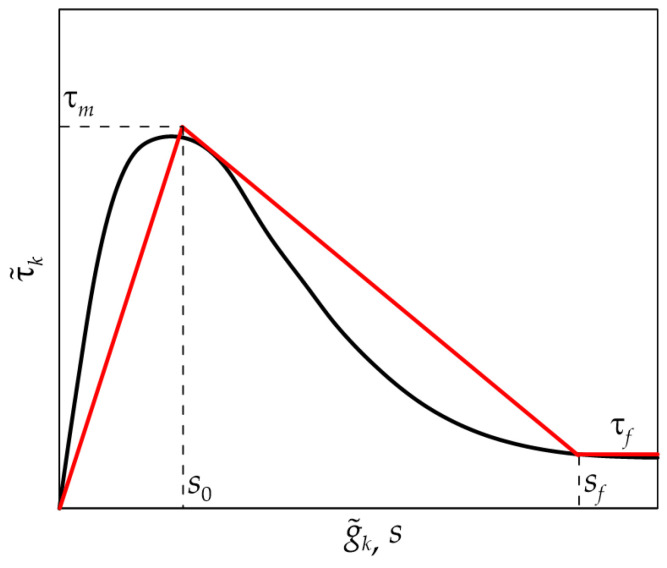
Comparison between the experimental ${\tilde{\tau }}_{k}$−${\tilde{g}}_{k}$ curve (in black) and the corresponding trilinear CML obtained with the procedure proposed (in red).

Figure 6 shows the trilinear CML obtained using the procedure proposed, compared with the experimental ${\tilde{\tau }}_{k}$−${\tilde{g}}_{k}$ curve (see Figure 4). In Figure 6, ${\tilde{\tau }}_{k_{fr}}$ and ${\tilde{g}}_{k_{fr}}$ were replaced with τ*_f_* and *s_f_*, respectively.

**Final checks**. To confirm that the calibrated trilinear CML correctly and accurately describes the experimental response, it can be substituted in Equation (16) to obtain the analytical load response to be compared with the corresponding experimental *P*–*g* relationship. However, since Equation (16) assumes infinite bonded length, the trilinear CML should be used to solve Equation (1) and compared with the experimental load response to assure that the free end slip can be neglected (see Step 5).

## 3. Results and Discussion

The procedure proposed was applied to obtain the CML that describes the matrix–fiber interface of various FRCM composites. Namely, the experimental load responses of PBO FRCM–concrete joints [23,33], carbon FRCM–masonry joints [34], glass FRCM–concrete joints [35], and glass FRCM–masonry joints [36] were considered. For each type of composite, the *P*–*g* responses obtained with two single-lap direct shear tests were analyzed. All composite strips applied either to a concrete block or to a masonry wallet included a single layer of textile except for two PBO FRCM–concrete joints [33], which included two layers of textile. The geometrical and mechanical properties of the textile and matrix comprising the composite strips are provided in Table 1, where *t_f_* = textile equivalent thickness, b^*^ = width of a single textile yarn, *f_f_* = textile tensile strength, *E_f_* = textile elastic modulus, *f_mu_* = matrix compressive strength [37], and *f_mt_* = matrix flexural strength [37].

The FRCM strips considered had different bonded lengths ℓ and widths *b*_1_, including a different number of longitudinal yarns *n*. Each specimen was named following the notation adopted in the corresponding publication. The geometrical properties of the FRCM strips of each specimen, including the number of layers *L* and the textile cross-sectional area *A_f_*, are provided in Table 2, along with the peak load attained P*.

**Figure 7:**
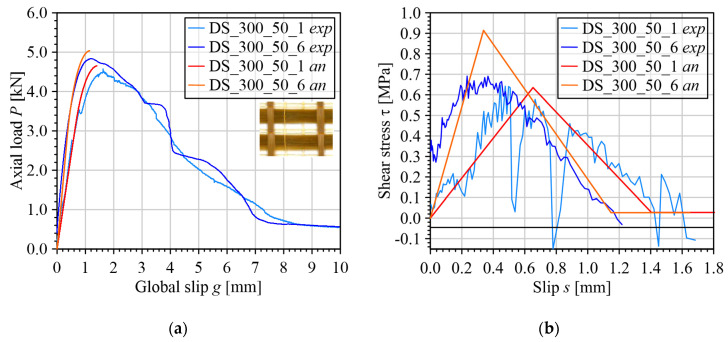

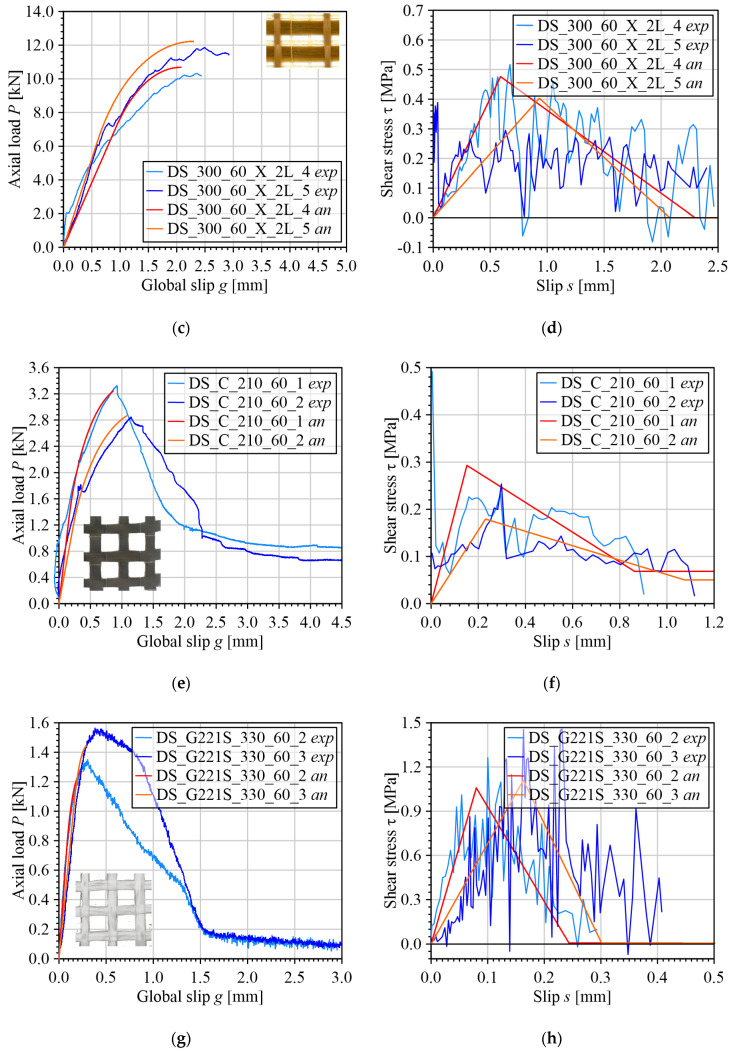

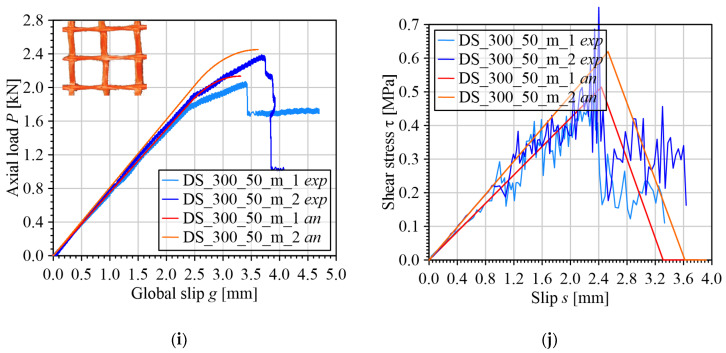
Comparison between analytical and experimental load responses and corresponding CML: (**a**,**b**) PBO FRCM–concrete joints; (**c**,**d**) PBO FRCM–concrete joints with two layers of textile; (**e**,**f**) carbon FRCM–masonry joints; (**g**,**h**) bare glass FRCM–concrete joints; (**i**,**j**) coated glass FRCM–masonry joints.

Figure 7 shows that, despite the irregularity of the experimental ${\tilde{\tau }}_{k}-{\tilde{g}}_{k}$ responses (due to the numerical differentiation of the experimental $P_{j}-g_{j}$ responses), the simple trilinear model allows for capturing the experimental *P*–*g* responses up to the onset of debonding for different FRCM composites.

Three main critical aspects can be identified in the proposed procedure. The first critical aspect is related to the determination of *s_f_*. This slip is defined in Step 5 as the minimum slip corresponding to the crossing of the horizontal line $\tau =\tau_{f}$ by the ${\tilde{\tau }}_{k}-{\tilde{g}}_{k}$ response. Due to its irregularity, the ${\tilde{\tau }}_{k}-{\tilde{g}}_{k}$ could cross the $\tau =\tau_{f}$ line at several slips, as happens in Figure 7b,d,h. In such cases, assuming that *s_f_* is located in the descending portion of the ${\tilde{\tau }}_{k}-{\tilde{g}}_{k}$ response, *s_f_* should be chosen so that for slips greater than *s_f_* the shear stress ${\tilde{\tau }}_{k}$ is similar to τ*_f_*. This is the reason why $s_{f}≅1.4\;\;\mathrm{mm}$ was chosen for specimen DS_300_50_1 instead of $s_{f}≅0.8\;\;\mathrm{mm}$. A rational criterion to establish whether the right value of *s_f_* has been identified consists of decreasing $\tilde{N}$, which entails for a smoother ${\tilde{\tau }}_{k}-{\tilde{g}}_{k}$ response, and checking if a similar *s_f_* is obtained.

The second critical aspect arises from the assumption that the experimental free end slip is zero. The correctness and eventually the influence of this assumption should be checked by comparing the analytical *P*(*g*) response obtained with Equation (16), which assumes zero slip at the free end, and the *P*(*g*) response obtained with the procedure described in [42], which is based on Equation (1) and allows for nonzero slip at the free end. The two *P*(*g*) responses should be consistent, at least up to $g=s_{f}$.

The third critical aspect arises from the assumption that the bonded length adopted in the experimental tests is greater than the effective bond length. If the bonded length of the FRCM composite considered is not known from previous work, it is necessary to apply the procedure with experimental results obtained with different bonded lengths and check that the obtained CMLs do not depend on the bonded length. If a dependency of the CML on the bonded length is found, it is possible that the short bonded lengths are shorter than the effective bond length. Consequently, the CMLs determined based on the *P*–*g* response of those specimens should be disregarded.

The results obtained confirmed that the proposed procedure can be effectively adopted to obtain the CML from the load response of direct shear tests, without the need for a direct measurement of the composite axial strain. Furthermore, the CML shape adopted provided a simple solution of the differential equation in Equation (1). Due to the complex behavior of FRCM–substrate joints, the procedure required a careful analysis of the load response obtained, since slight variations in the CML can be obtained by varying, for instance, the parameters considered to reduce the oscillations in the load response (see Step 1). However, the final checks proposed allow for verifying that the CML calibrated correctly reproduces the experimental results.

## 4. Conclusions

In this paper, an analytical procedure to determine a trilinear CML of FRCM–substrate joints was applied to carbon, AR glass, and PBO FRCM composites applied to concrete and masonry substrates. The results obtained allowed for drawing the following main conclusions:

- The proposed procedure may be used to estimate the parameters of a trilinear CML able to accurately reproduce the experimental load response. Attention should be paid in determining the parameters needed for the procedure. However, the accuracy of the procedure can be assessed by comparing the analytical load response provided by the calibrated CML with the experimental load response.
- The proposed procedure represents a valuable tool to estimate the CML of FRCM–substrate joints that can then be used to identify fundamental features of the FRCM composite, such as the onset of debonding in FRCM–substrate joints, the crack number and spacing in FRCM coupons, and the locations where debonding occurs in FRCM-strengthened members.
- The proposed procedure allows for simply and rapidly obtaining the parameters of the trilinear CML, which can be used in nonlinear finite element models to estimate the behavior of concrete or masonry structural members strengthened with FRCM composites.

## Figures and Tables

**Table 1:** Geometrical and mechanical properties of the textile and matrix comprising the composites.

Composite	*t_f_* [mm]	b* [mm]	*f_f_* [MPa]	*E_f_* [GPa]	*f_mu_* [MPa]	*f_mt_* [MPa]
PBO FRCM–concrete	0.046 [38]	5.0 [38]	3014 [25]	206 [25]	51.6 [23]	8.1 [23]
Carbon FRCM–masonry	0.094 [39]	5.0 [39]	1944 [34]	203 [34]	25.0 [34]	6.1 [34]
Glass FRCM–concrete	0.048 [35]	4.0 [35]	1300 [35]	-	35.5 [35]	6.1 [35]
Glass FRCM–masonry	0.063 [40]	2.7 [40]	756 [36]	52 [36]	22.0 ^1^ [41]	6.0 ^1^ [41]

^1^ Declared by the manufacturer [41].

**Table 2:** Geometrical and mechanical properties of the textile and matrix comprising the composites.

Composite	Name	ℓ [mm]	*b*_1_ [MPa]	*n* [-]	*L* [-]	*A_f_* [mm^2^]	P* [kN]
PBO FRCM–concrete	DS_300_50_1	300	50	5	1	2.30	4.58
	DS_300_50_6	300	50	5	1	2.30	4.84
	DS_300_60_2L_X_4	300	60	6	2	5.52	10.32
	DS_300_60_2L_X_5	300	60	6	2	5.52	11.85
Carbon FRCM–masonry	DS_C_210_60_1	210	60	6	1	5.64	3.32
	DS_C_210_60_2	210	60	6	1	5.64	2.85
Glass FRCM–concrete	DS_G221S_330_60_2	330	60	5	1	2.88	1.35
	DS_G221S_330_60_3	330	60	5	1	2.88	1.56
Glass FRCM–masonry	DS_300_50_c_1	300	50	3	1	3.15	2.03
	DS_300_50_c_2	300	50	3	1	3.15	1.98

## Data Availability

Data are contained within the article.
